# Pilot Evaluation of a Web Application for Amblyopia Risk Screening Integrating Parent-Reported Factors with AI-Assisted Strabismus Detection

**DOI:** 10.22599/bioj.493

**Published:** 2026-02-27

**Authors:** Mustapha Jaouhari, Chaimae El Harrak, Farida Bentayeb, Youssef Elmerabet

**Affiliations:** 1 Laboratory of Electronic, Mechanical, and Energetic Information Processing Systems, Faculty of Sciences, Ibn Tofail University, Morocco; 2 Laboratory of Engineering and Materials (LIMAT), Hassan II University of Casablanca, Faculty of Sciences Ben M’Sick, Morocco

**Keywords:** Amblyopia screening, Artificial intelligence, Web-based tool, Paediatric vision, Strabismus detection, School screening, Preventive eye care

## Abstract

**Background:**

Amblyopia is the most common cause of visual impairment in children, and early detection is essential, yet screening remains limited in many settings, especially where access to eye-care specialists is scarce.

**Objective:**

To evaluate the accuracy of a web-based screening tool that combines parent-reported risk factors with AI-assisted strabismus detection for identifying children at risk of amblyopia.

**Methods:**

This pilot study included 105 children aged 3–10 years attending a public hospital in Morocco for their first ophthalmological evaluation. Parents completed an online screening tool consisting of eight validated amblyopia risk-factor questions and an automated strabismus analysis based on a frontal smartphone photograph. The AI module combined geometric measurements of pupil–nasal root symmetry with convolutional neural network (CNN) features such as corneal light reflex and gaze vector orientation. Each child received a total score (0–9), stratified into high-risk (6–9), moderate-risk (3–6), or low-risk (0–3) categories. A comprehensive ophthalmological examination, performed by a clinician blinded to the application results, served as the reference standard.

**Results:**

Of the 105 children screened, 32 were classified as high-risk, 62 as moderate-risk, and 11 as low-risk. The tool demonstrated perfect agreement in the high-risk category, with all 32 high-risk children clinically confirmed to have amblyopia (PPV = 100%). In the moderate-risk group, 30 of the 62 children were clinically confirmed (PPV = 48.4%). No child in the low-risk group had amblyopia (NPV = 100%). The AI-assisted strabismus module showed strong predictive accuracy in the high-risk category (96.9% confirmation). Statistical analyses showed no significant differences in diagnostic performance across age, gender, or urban/rural subgroups (p > 0.05).

**Conclusions:**

The hybrid screening tool reliably identified children at high risk for amblyopia with complete concordance with a blinded clinical diagnosis, while safely excluding low-risk children. Although moderate-risk scores require cautious interpretation and clinical follow-up, this approach offers a low-cost, accessible, and scalable solution for paediatric vision screenings in resource-limited settings. Further large-scale community-based studies are warranted to validate generalisability.

## Introduction

Amblyopia remains the most common cause of unilateral visual impairment in children, affecting approximately 2–4% of the paediatric population worldwide (Holmes and Clarke, 2006). Early detection is essential, as timely intervention during the critical period of visual development significantly improves treatment outcomes and reduces the long-term impact on binocular vision and academic performance. Despite this, access to systematic screening remains limited in many low- and middle-income settings, where shortages of trained eyecare professionals and the absence of school-based programmes contribute to delayed diagnosis (Donahue et al., 2016). Innovative and scalable screening strategies are therefore urgently needed to support early identification of children at risk.

Recent technological advances have enabled the development of digital tools capable of assisting with visual screening outside traditional clinical environments. Automated detection of ocular misalignment from photographs, for example, has demonstrated promising accuracy and offers a low-cost method for identifying strabismus, one of the leading risk factors for amblyopia (Hunter et al., 2003). Combining such automated analysis with validated parental questionnaires may offer a practical, accessible, and cost-effective alternative to conventional screening, particularly in resource-constrained contexts.

In this pilot study, we evaluate a web-based application that integrates parent-reported amblyopia risk factors with AI-assisted strabismus detection from smartphone images. The objective is to assess whether this hybrid tool can reliably identify children at risk of amblyopia when compared with a blinded, gold-standard ophthalmological examination and to explore its potential as a scalable approach for community-level screening.

## Methods

This pilot study was conducted in the outpatient paediatric department of a public hospital in Morocco and included approximately 105 children aged 3–10 years recruited consecutively after informed consent from parents or legal guardians. Although a small number of children had previously received eye care or wore spectacles prescribed elsewhere, all participants were attending the ophthalmology service of the participating hospital for the first time, and neither the clinical team nor the examining ophthalmologist had access to prior diagnostic records within this institution. However, we acknowledge that some children had previously received eye care elsewhere and/or were already wearing spectacles, meaning that the cohort cannot be considered truly naïve.

As a feasibility pilot study, hospital-based sampling was intentionally chosen to allow controlled conditions and immediate access to a gold-standard ophthalmological examination. While examiner blinding to prior diagnoses within the hospital and to application results was implemented, complete masking could not be ensured due to visible indicators such as spectacle wear and parental awareness. This potential source of selection and information bias is therefore acknowledged as a limitation of the study.

The web-based screening was performed before the clinical assessment, and an essential methodological safeguard was implemented to preserve the integrity of the comparison: the ophthalmologist performing the examination was fully blinded to the application results, and parents were explicitly instructed not to disclose the screening outcome to the clinician. Parents were thoroughly informed about the importance of maintaining this blinding process, and many participated actively and responsibly, effectively acting as collaborators to ensure that the evaluation remained unbiased. Several parents later shared their impressions regarding whether the application had correctly identified their child’s visual difficulty, but only after the clinician had completed the blind examination.

The screening itself used the web-based tool, in which parents uploaded a frontal smartphone photograph of the child and completed eight validated amblyopia risk-factor questions. The AI model processes images captured using any smartphone camera (iPhone, Samsung, or other devices capable of supplying a frontal face image). After image upload, the system detects facial landmarks, localises both pupils and the nasal root landmark, and calculates the Euclidean distance between each pupil centre and the nasal root. Under normal ocular alignment, these distances are expected to be symmetrical; asymmetry exceeding a predefined threshold is flagged as potential strabismus. This geometric assessment is combined with features extracted by a Convolutional Neural Network (CNN), including corneal light reflex symmetry and gaze vector orientation. A positive strabismus detection contributes one point to the total score, while each positive questionnaire item contributes one point, yielding a 0–9 total amblyopia-risk score.

Following the web-based assessment, all children underwent a comprehensive ophthalmological evaluation, including visual acuity measurement, cycloplegic refraction, ocular alignment testing, and fundus examination, performed independently and in complete blindness to the application results. This design allowed us to rigorously determine whether the web application produced results consistent with an unbiased gold-standard ophthalmological diagnosis, which is the primary aim of this pilot study.

**Figure 1:**
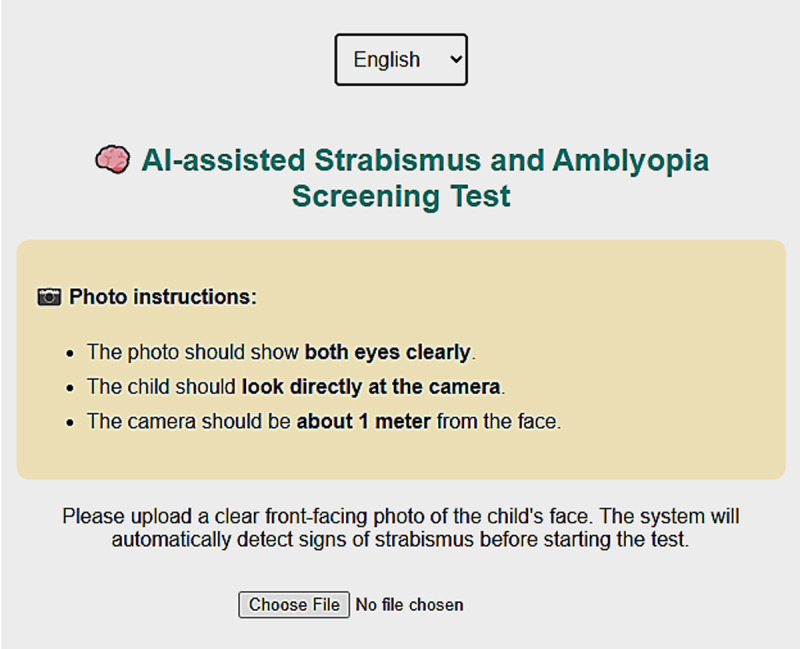
First page of the AI-powered web application. It includes a language selector, photo instructions.

At the beginning of the platform, users are presented with a language selection menu at the top of the homepage, enabling them to choose between Arabic and French or English for better accessibility (Figure 1).

After choosing the preferred language, users are guided to a page that includes clear, step-by-step photo instructions. These instructions request the parent or guardian to take or upload a frontal photo of the child’s face, ensuring that both eyes are clearly visible, the child is looking directly at the camera, and the camera is positioned about one `metre from the child’s face. An image upload field allows the user to submit the photo to the system, which then uses AI to analyse ocular alignment and detect signs of strabismus, one of the key risk factors for amblyopia. If strabismus is detected, 1 point is added to the child’s risk score.

Immediately after the AI image analysis, the application presents a brief questionnaire composed of eight yes/no questions, each addressing a validated amblyopia risk factor. Each ‘yes’ response is assigned one point, contributing to the final score. These questions are adapted from existing paediatric vision screening tools and supported by clinical evidence.

**The 8 Questions:**

1. Does your child squint or close one eye while focusing on something? (Holmes and Clarke, 2006)
2. Have you noticed your child rubbing their eyes frequently? (Rosenfield, 2011)
3. Does your child sit very close to the television or hold objects too close? (American Optometric Association, 2017)
4. Has your child ever failed a vision screening at school or daycare? (Donahue et al., 2016)
5. Do you have a family history of amblyopia, strabismus, or high refractive errors? (Simons, 2005)
6. Has your child complained of headaches or eye strain after reading or using screens? (von Noorden and Campos, 2002)
7. Does your child tilt or turn their head while looking at objects? (Kushner, 1998)
8. Do your child’s eyes appear misaligned in photos (one eye drifting inward or outward)? (Hunter et al., 2003)

### Scoring and follow-up

**Figure 2:**
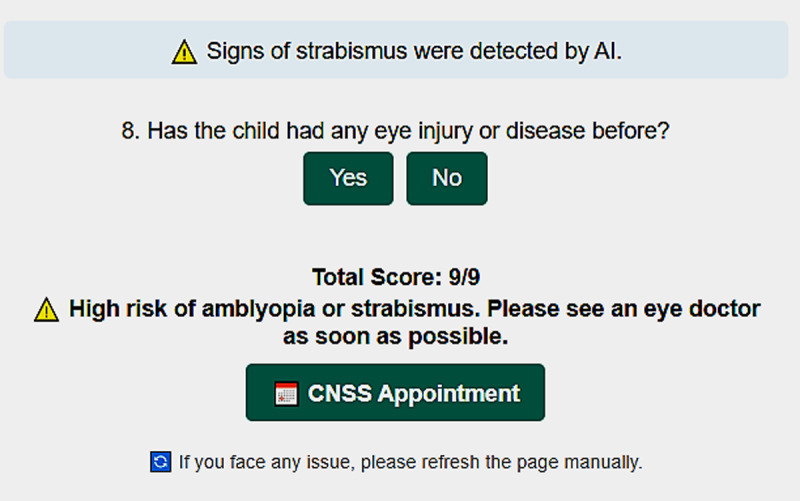
Final page of the AI-powered web application. It displays the child’s total risk score.

Each participant receives a total score out of nine points: one point derived from the AI-based strabismus analysis and eight points from the parent-reported questionnaire. Based on this total score, three mutually exclusive risk categories are defined: 0–3 points indicate a low risk of amblyopia, 4–6 points indicate a moderate risk, and 7–9 points indicate a high risk. At the end of the test, the system automatically displays the child’s risk category and, when applicable, explicitly indicates the AI-detected signs of strabismus in the uploaded photograph. The platform also presents the final score (e.g., ‘Total Score: 9/9’) together with a recommendation to seek ophthalmological evaluation, particularly for children in the high-risk group. To facilitate timely follow-up, a dedicated ‘CNSS Appointment’ button allows parents to directly access the official Moroccan Ministry of Health system and schedule an eye-care consultation (see Figure 2).

The web application is shared directly with the child’s parent or guardian via a smartphone (through a web link or QR code), allowing them to access the platform easily.

The AI-powered screening tool used in this study is accessible via a secure web interface at [https://ia-amblyopia.netlify.app/]. Users begin by selecting a language, uploading a photo for strabismus detection, and answering eight validated questions.

### Data analysis

Data were analysed using SPSS. Descriptive statistics were used to summarise demographic characteristics and distribution across the three amblyopia-risk categories (high, moderate, low). Diagnostic performance of the web-based screening tool was evaluated by calculating sensitivity, specificity, positive predictive value (PPV), and negative predictive value (NPV), using the blinded ophthalmological diagnosis as the gold standard.

### Ethical Considerations

This study received ethical clearance from the Biomedical Research Ethics Committee of Oujda (CERBO) and was conducted in accordance with the ethical principles outlined in the Declaration of Helsinki. Official authorisation was also obtained from the Moroccan Ministry of Health and Social Protection (approval number 2324/2025). Prior to enrolment, detailed information regarding the study’s objectives, procedures, and voluntary nature was provided to all participants and their guardians. Written informed consent was obtained from each child’s legal guardian, and assent was sought from the children in an age-appropriate manner. To protect privacy, all collected data were anonymised and stripped of personally identifiable information. The study was conducted in the Marrakech-Safi region at a provincial hospital that serves both urban and rural populations.

## Results

A total of 105 children (66 females and 39 males) aged between 3 and 10 years participated in the pilot study. Among them, 23 children were aged 3–5 years, 52 were 5–8 years, and 30 were 8–10 years. The sample included 70 children from urban areas and 35 from rural areas, and seven children (6.7%) were already wearing spectacles at the time of screening, while the remaining 98 children (93.3%) had no prior optical correction. All participants were screened using the AI-assisted web application and subsequently examined by a blinded ophthalmologist.

Based on the application’s scoring system, 32 children (30.5%) were classified in the high-risk category (score 7–9), 62 (59.0%) in the moderate-risk category (score 4–6), and 11 (10.5%) in the low-risk category (score 0–3). The ophthalmological examination confirmed amblyopia in all children categorised as high-risk (32/32; PPV = 100%), whereas 30 of the 62 moderate-risk children were confirmed to have the condition (PPV = 48.4%). None of the 11 children in the low-risk category were found to have amblyopia (NPV = 100%).

Strabismus detection by the AI component showed similarly strong performance in the high-risk group, detecting 32 cases with 31 confirmed clinically (PPV = 96.9%). In the moderate-risk group, the AI detected 30 cases of ocular misalignment, with 28 confirmed by the ophthalmologist (PPV = 93.3%). Age-stratified analysis showed no statistically significant association between age group and screening outcome (p = 0.3382, Fisher-Freeman-Halton test). Diagnostic accuracy remained high across ages, with perfect a PPV (100%) for high-risk children in all age strata and a 100% NPV for all low-risk groups. Gender-based analysis also revealed no significant difference in detection performance (p = 0.5537, Fisher’s exact test). Among males, the PPV for moderate-risk scores was 47.5%, whereas among females it was 28.9%, with both groups showing perfect prediction for high-risk and low-risk categories. Likewise, no significant association was found between urban versus rural residence and screening accuracy (p = 1.0000). High-risk detection demonstrated PPV values of 100% in both settings, and NPV remained 100% in all low-risk subgroups (Table 1).

**Table 1:** Performance of the AI-based web application for amblyopia screening compared to clinical diagnosis across demographic and risk groups.


CATEGORY	GROUP CATEGORY BY THE APP	AMBLYOPIA CONFIRMED (OPH.)	STRABISMUS DETECTED BY APP (N)	STRABISMUS CONFIRMED BY OPH. (N)	ACCURACY	PPV	NPV	P-VALUE (TEST USED)

Score Category	High risk (6–9) n = 32	32	32 (19 Exotropia – 13 Esotropia)	31 (18 Exotropia –13 Esotropia)	100%	100%	—	—

	Moderate risk (3–6) n = 62	30	30 (19 Exotropia – 9 Esotropia)	28 (20 Exotropia – 10 Esotropia)	48.4%	48.4%	—	—

	Low risk (0–3) n = 11	0	0	0	100% NPV	—	100%	—

**Age 3–5**	High risk: 8	High risk: 8	High risk: 8	High risk: 7	—	100%	—	0.3382 (Chi-Square Test)

	Moderate risk: 12	Moderate risk: 4	Moderate risk: 8	Moderate risk: 6	—	33.3%	—	0.3382 (Chi-Square Test)

	Low risk: 6	Low risk: 0	Low risk: 0	Low risk: 0	—	—	100%	0.3382 (Chi-Square Test)

**Age 5–7**	High risk: 10	High risk: 10	High risk: 10	High risk: 10	—	100%	—	0.3382 (Chi-Square Test)

	Moderate risk: 16	Moderate risk: 7	Moderate risk: 8	Moderate risk: 8	—	43.8%	—	0.3382 (Chi-Square Test)

	Low risk: 3	Low risk: 0	Low risk: 0	Low risk: 0	—	—	100%	0.3382 (Chi-Square Test)

**Age 7–10**	High risk: 14	High risk: 14	High risk: 14	High risk: 14	—	100%	—	0.3382 (Chi-Square Test)

	Moderate risk: 34	Moderate risk: 19	Moderate risk: 14	Moderate risk: 14	—	41.1%	—	0.3382 (Chi-Square Test)

	Low risk: 2	Low risk: 0	Low risk: 0	Low risk: 0	—	—	100%	0.3382 (Chi-Square Test)

**Gender – Male**	High risk: 10	High risk: 10	High risk: 10	High risk: 9	—	100%	—	0.5537 (Fisher’s Exact Test)

	Moderate risk: 24	Moderate risk: 19	Moderate risk: 16	Moderate risk: 14	—	47.5%	—	0.5537 (Fisher’s Exact Test)

	Low risk: 5	Low risk: 0	Low risk: 0	Low risk: 0	—	—	100%	0.5537 (Fisher’s Exact Test)

**Gender – Female**	High risk: 22	High risk: 22	High risk: 22	High risk: 22	—	100%	—	0.5537 (Fisher’s Exact Test)

	Moderate risk: 38	Moderate risk: 11	Moderate risk: 14	Moderate risk: 12	—	28.9%	—	0.5537 (Fisher’s Exact Test)

	Low risk: 6	Low risk: 0	Low risk: 0	Low risk: 0	—	—	100%	0.5537 (Fisher’s Exact Test)

**Area – Urban**	High risk: 22	High risk: 22	High risk: 22	High risk: 21	—	100%	—	1.0000 (Fisher’s Exact Test)

	Moderate risk: 42	Moderate risk: 12	Moderate risk: 18	Moderate risk: 16	—	28.6%	—	1.0000 (Fisher’s Exact Test)

	Low risk: 4	Low risk: 0	Low risk: 0	Low risk: 0	—	—	100%	1.0000 (Fisher’s Exact Test)

**Area – Rural**	High risk: 10	High risk: 10	High risk: 10	High risk: 10	—	100%	—	1.0000 (Fisher’s Exact Test)

	Moderate risk: 20	Moderate risk: 18	Moderate risk: 12	Moderate risk: 12	—	60%	—	1.0000 (Fisher’s Exact Test)

	Low risk: 5	Low risk: 0	Low risk: 0	Low risk: 0	—	—	100%	1.0000 (Fisher’s Exact Test)


Overall, the AI-assisted web tool demonstrated excellent performance in identifying children at high risk of amblyopia and strabismus, with perfect agreement between the application’s high-risk classification and the blinded ophthalmological diagnosis. Moderate-risk scores showed partial predictive value, indicating that this group remains clinically relevant and warrants systematic follow-up. Low-risk scores demonstrated complete agreement with the absence of amblyopia on clinical examination. Importantly, no demographic variable, including age, gender, or area of residence, showed a statistically significant association with diagnostic accuracy, suggesting that the tool performs consistently across different subpopulations.

## Discussion

This pilot study evaluated the performance of a web-based amblyopia risk-screening tool that combines parent-reported visual behaviour indicators with an AI-assisted strabismus detection module. The results demonstrate that the application achieved excellent diagnostic accuracy in identifying children at high risk of amblyopia, with a perfect concordance between the high-risk category and the ophthalmological diagnosis (PPV = 100%). These findings are consistent with previous literature showing that risk-factor-based screening approaches are highly effective when clear behavioural signs or family-reported symptoms are present (Simons, 2005).

The tool also performed strongly in detecting ocular misalignment, with the AI module achieving 96.9% confirmation in the high-risk group. This aligns with prior work indicating that automated strabismus analysis using geometric calculations and convolutional neural networks can achieve high sensitivity when images are well-centred and stable (Yarkheir et al., 2025). Importantly, the few misclassified cases were found exclusively among children aged 3–5 years. This age group is known to pose challenges for automated detection due to difficulty maintaining stable fixation, as well as age-specific facial morphology, particularly epicanthal folds, which can mimic pseudo-strabismus and lead to inaccuracies in corneal reflex symmetry and pupil nasal root distance measurements (Birch and Holmes, 2010). These findings suggest that future refinements may require age-adjusted calibration parameters or additional morphological correction factors to optimise performance in younger children.

Performance in the moderate-risk group was markedly lower (PPV = 48.4%), which is consistent with the known complexity of identifying children with mild or borderline risk factors. In epidemiological studies, moderate-risk categories often include children with uncorrected refractive errors or mild visual symptoms that do not meet the full clinical criteria for amblyopia (Chen and Cotter, 2016). This suggests that the moderate-risk classification remains clinically meaningful but requires systematic ophthalmological follow-up rather than serving as a definitive diagnostic indicator. The complete absence of amblyopia among low-risk children (NPV = 100%) further supports the safety and reliability of the tool for excluding children classified in the lowest risk category.

Although the examining ophthalmologist was fully blinded to the application results, we acknowledge that the study population cannot be considered truly naïve, as a subset of children had previously received eye care and/or were wearing spectacles prescribed elsewhere.

Examiner blinding was implemented to reduce expectation bias at the level of clinical assessment; however, visible indicators such as spectacle wear and parental awareness may still have influenced responses and observations. This potential source of bias is therefore acknowledged as a limitation. Nevertheless, maintaining examiner blinding to digital screening outcomes remains an important methodological safeguard in digital health validation studies, as recommended in the literature (Garg et al., 2016) and by the American Association for Paediatric Ophthalmology and Strabismus for paediatric vision screening research.

Diagnostic performance was highly consistent across demographic subgroups, including age (outside the 3–5 subgroup), gender, and geographical area, with no statistically significant differences detected. This uniformity suggests that the tool is equitable and accessible across diverse populations, including rural communities, an important consideration for large-scale implementation in low-resource settings.

Despite these encouraging results, several limitations require consideration. This was a hospital-based pilot, and the prevalence of amblyopia and strabismus in such settings is naturally higher than in school-based populations. Larger community-based studies will be required to determine real-world performance in asymptomatic children. The moderate-risk group’s modest predictive value highlights the need for refinement, potentially through enhanced AI integration such as the addition of photorefraction analysis, automated pupillary asymmetry metrics, or deeper CNN-based feature extraction capable of capturing subtle ocular characteristics. Additionally, this study assessed only immediate diagnostic accuracy, not long-term impact on referral patterns, treatment adherence, or visual outcomes. These aspects will be important in future implementation research.

Overall, the results of this pilot study provide strong evidence that a low-cost, accessible, smartphone-based application integrating parental reporting with AI-assisted strabismus analysis can serve as a reliable initial screening tool in resource-limited environments. The high predictive accuracy for high-risk cases, combined with the perfect negative predictive value in low-risk children, underscores the tool’s potential for large-scale deployment. These findings support further development and community-based validation to establish broader applicability and to refine AI performance in younger age groups.

## Conclusion

This pilot study shows that the web-based tool combining parent-reported risk factors with AI-assisted strabismus detection can accurately identify children at high risk of amblyopia, with excellent agreement with blinded clinical diagnosis. The tool performed consistently across demographic groups and reliably excluded low-risk children. Moderate-risk scores, while less specific, still identified a substantial proportion of true cases and therefore warrant clinical follow-up. These findings suggest that this low-cost hybrid approach may support early detection in resource-limited settings, although larger community-based studies are needed to confirm its broader applicability.
